# Coacervate‐Derived Hydrogel with Effective Water Repulsion and Robust Underwater Bioadhesion Promotes Wound Healing

**DOI:** 10.1002/advs.202203890

**Published:** 2022-09-15

**Authors:** Xin Peng, Yuan Li, Tianjie Li, Yucong Li, Yingrui Deng, Xian Xie, Yi Wang, Gang Li, Liming Bian

**Affiliations:** ^1^ Molecular Imaging Center Guangdong Provincial Key Laboratory of Biomedical Imaging The Fifth Affiliated Hospital Sun Yat‐sen University Zhuhai 519000 P. R. China; ^2^ Department of Orthopaedics and Traumatology Stem Cells and Regenerative Medicine Laboratory Li Ka Shing Institute of Health Sciences The Chinese University of Hong Kong Prince of Wales Hospital Shatin Hong Kong SAR 999077 P. R. China; ^3^ Department of Physics The Chinese University of Hong Kong Hong Kong SAR 999077 P. R. China; ^4^ Department of Biomedical Engineering The Chinese University of Hong Kong Hong Kong SAR 999077 P. R. China; ^5^ School of Biomedical Sciences and Engineering Guangzhou International Campus South China University of Technology Guangzhou 511442 P. R. China; ^6^ National Engineering Research Center for Tissue Restoration and Reconstruction South China University of Technology Guangzhou 510006 P. R. China; ^7^ Guangdong Provincial Key Laboratory of Biomedical Engineering South China University of Technology Guangzhou 510006 P.R. China; ^8^ Key Laboratory of Biomedical Materials and Engineering of the Ministry of Education South China University of Technology Guangzhou 510006 P. R. China

**Keywords:** coacervate, interfacial water, underwater adhesion, water repulsion, wound healing

## Abstract

Achieving robust underwater adhesion by bioadhesives remains a challenge due to interfacial water. Herein a coacervate‐to‐hydrogel strategy to enhance interfacial water repulsion and bulk adhesion of bioadhesives is reported. The polyethyleneimine/thioctic acid (PEI/TA) coacervate is deposited onto underwater substrates, which can effectively repel interfacial water and completely spread into substrate surface irregularities due to its liquid and water‐immiscible nature. The physical interactions between coacervate and substrate can further enhance interfacial adhesion. Furthermore, driven by the spontaneous hydrophobic aggregation of TA molecules and strong electrostatic interaction between PEI and TA, the coacervate can turn into a hydrogel in situ within minutes without additional stimuli to develop enhanced matrix cohesion and robust bulk adhesion on diverse underwater substrates. Molecular dynamics simulations further reveal atomistic details of the formation and wet adhesion of the PEI/TA coacervate via multimode physical interactions. Lastly, it is demonstrated that the PEI/TA coacervate‐derived hydrogel can effectively repel blood and therefore efficiently deliver the carried growth factors at wound sites, thereby enhancing wound healing in an animal model. The advantages of the PEI/TA coacervate‐derived hydrogel including body fluid‐immiscibility, strong underwater adhesion, adaptability to fit irregular target sites, and excellent biocompatibility make it a promising bioadhesive for diverse biomedical applications.

## Graphical abstract

Coacervate deposited onto underwater or wet substrates can effectively repel interfacial water and completely spread into substrate surface irregularities, resulting in tight contact and interfacial adhesion, due to its liquid and water‐immiscible nature. Then, driven by the spontaneous hydrophobic aggregation, coacervate can turn into a hydrogel in situ without additional stimuli to develop enhanced matrix cohesion and robust bulk adhesion.

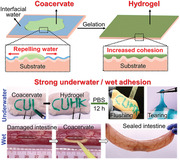

## 1 Introduction

Adhesive hydrogels have been considered as the promising biodhesives for wound dressings,^[^ ^1^ ^]^ hemostatic materials,^[^ ^2^ ^]^ sealants,^[^ ^3^ ^]^ wearable devices,^[^ ^4^ ^]^ and drug carriers.^[^ ^5^ ^]^ The adhesion strength of hydrogels on substrates depends on both the interfacial adhesion and the cohesion of hydrogels. Due to physical obstruction and dilution, the ample body fluids present on the surfaces of biological tissues can significantly hamper the establishment of tight and congruent hydrogel–tissue contact by the prefabricated hydrogels and hydrogels formed in situ via cross‐linked precursor solutions. Moreover, the seamless attachment of prefabricated adhesive hydrogels with fixed shape can be further compromised by the irregular tissue surface topography.^[^ ^6^ ^]^ Therefore, developing new strategies to prepare adhesive hydrogels that can form strong adhesion on wet tissues with irregular surfaces is highly desired.

A previous study demonstrated that the hydrogels containing biomimetic micro‐ or nanostructure arrays exhibit immediate, strong, and reversible adhesion under wet and underwater conditions due to the water‐absorbing properties and capillary adhesion of the microstructured hydrogel.^[^ ^7^ ^]^ Inspired by the bioadhesives secreted by some marine organisms such as sandcastle worms and mussels, recent works demonstrated that coacervates obtained via liquid–liquid phase separation can form tight interfacial adhesion on wet and underwater substrates.^[^ ^8^ ^]^ Due to their unique properties such as fluidity, water‐immiscibility, and low surface tension, coacervates can effectively repel interfacial water, spread on tissue surfaces, and fill up surface irregularities, thereby forming tight contact with substrates. Meanwhile, the functional groups in coacervate and substrate can form chemical or/and physical bonds to induce tight interfacial adhesion. Recently, Cui et al. prepared a hyperbranched polymer adhesive (HBPA) consisted of hydrophobic backbones and catechol‐bearing hydrophilic branches. Upon contacting water, phase transition and coacervation of HBPA can repel the interfacial water and expose catechol groups to enhance interfacial adhesion via the formation of physical and chemical bonds with substrates.^[^ ^2b^ ^]^ Nevertheless, the fluidity and low cohesion of coacervates limit the development of robust bulk adhesion.

For the bioadhesives secreted by sandcastle worms and mussels, minerals^[^ ^9^ ^]^ and enzyme^[^ ^10^ ^]^ can increase cross‐linking of amino acids (such as phosphoserine, 3,4‐dihydroxyphenylalanine, lysine, and cysteine), and this promotes the transition of liquid coacervates to cross‐linked materials with increased cohesion and bulk adhesion to substrates. However, only very few works have attempted to increase the cohesion of coacervate to realize strong bulk adhesion. Narayanan et al. prepared a hybrid protein‐like polyester (HyPPo) coacervate made of tropoelastin‐mimetic domains for coacervation, catechol groups for interfacial adhesion, and pendant coumarin groups for cohesion. The UV‐triggered cross‐linking of coumarin groups in HyPPo increased the cohesion of coacervate, resulting in strong adhesion on substrates.^[^ ^11^ ^]^ Therefore, applying a coacervate to establish tight substrate contact first followed by elevating the coacervate cohesion provides a new approach to enhancing the bulk adhesion of bioadhesives.

Thioctic acid (TA), which contains hydrophobic 1,2‐dithiolanes and alkyl chain groups as well as hydrophilic carboxylic acid groups, is a naturally existing small molecule and acts as an essential coenzyme for aerobic metabolism in animals. TA can form two types of noncovalent bonds, hydrophobic aggregation of 1,2‐dithiolanes and alkyl chain groups, and electrostatic interaction or hydrogen bonding of carboxylic acid groups. This unique molecular structure makes TA a potential candidate for forming complex coacervate through self‐assembling.^[^ ^12^ ^]^ However, TA is insoluble in water, it is difficult to directly form a complex with other water‐soluble polymers in aqueous solution. Branched polyethylene imine (PEI) containing abundant amine groups with almost no steric hindrance has been used as gene carrier material because of its capability of forming stable complexes through electrostatic interactions with nucleic acids.^[^ ^13^ ^]^ The unique chemical structure of branched PEI can help its complexation with TA. On the one hand, the abundant amine groups with minimal steric hindrance in PEI can form strong electrostatic interaction with the carboxylic acid groups in TA. On the other hand, branched PEI structure can also facilitate the formation of electrostatic interactions between PEI and TA and hydrophobic interactions between TA molecules. Therefore, branched PEI can efficiently complex with TA to effectively enhance TA solubility in aqueous solution.

Herein, we report a PEI/TA coacervate‐derived hydrogel, which can achieve robust bulk adhesion on various wet and underwater substrates. The PEI/TA coacervate deposited on wet and underwater substrates can effectively repel interfacial water and fill up the substrate surface irregularities, thereby forming tight contact with the substrates. Meanwhile, the functional groups between coacervate and substrate can form physical bonds to induce tight interfacial adhesion. The spontaneous aggregation of hydrophobic 1,2‐dithiolanes of TA molecules enhances the cross‐linking and the cohesion of the coacervate, resulting in the coacervate–hydrogel transition in situ without any additional stimuli. Therefore, the coacervate‐derived hydrogel can develop robust bulk adhesion on various substrates. We further demonstrate that the PEI/TA coacervate‐derived hydrogel loaded with epidermal growth factor (EGF) as wound dressing can effectively repel blood and seamlessly adhere to wound site tissues, thereby enhancing the delivery of EGF. The advantages of PEI/TA coacervate‐derived hydrogel including strong wet adhesion, body fluid‐immiscibility, adaptability to fit irregularly‐shaped target sites, easy preparation and usage, and excellent cytocompatibility make it a promising adhesive for diverse biomedical applications.

## 2 Results and Discussion

### 2.1 Preparation of PEI/TA Coacervate and Coacervate‐Derived Hydrogel

**Figure 1:**
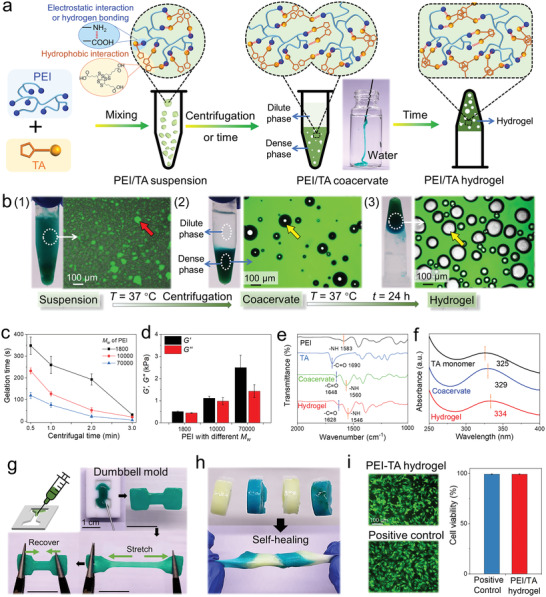
Preparation of polyethyleneimine/thioctic acid (PEI/TA) coacervate and coacervate‐derived hydrogel. a) Schematic illustration of the formation of PEI/TA coacervate and coacervate‐derived hydrogel. b) Transition of PEI/TA complex suspension to coacervate and coacervate‐derived hydrogel (red arrow: coacervate droplets, yellow arrow: vacuoles). The fluorescent region is PEI_FITC_/TA complex. Macrographs are the merge of bright and fluorescent images. c) Gelation time of PEI/TA coacervate prepared from PEI of different *M*_w_. Test conditions: 37 °C in PBS (*n* = 3). d) Storage modulus (*G′*) and loss modulus (*G″*) of cylindrical PEI/TA coacervate‐derived hydrogels prepared from PEI of different *M*_w_ after gelation at 37 °C for 3 min (*n* = 3). e) FTIR spectra of the PEI, TA, PEI/TA coacervate, and PEI/TA coacervate‐derived hydrogel. f) UV–vis spectra of the TA monomer, PEI/TA coacervate, and PEI/TA coacervate‐derived hydrogel. g) Injection of PEI/TA coacervate into a custom model for the fabrication of hydrogel. h) Self‐healing of PEI/TA coacervate‐derived hydrogel. i) Live/dead staining (left) and cell viability (right) of 3T3 cells after being coincubated with PEI/TA hydrogel or positive control media for 24 h (*n* = 3). Data are shown as the mean ± SD.

The tandem formation of polyethyleneimine/thioctic acid (PEI/TA) coacervate and coacervate‐derived hydrogel can be divided into three steps (**Figure** **1**a,b). To clearly observe the formation of PEI/TA coacervate and coacervate‐derived hydrogel, the PEI molecules were labeled with fluorescein isothiocyanate (FITC). First, by mixing 220 mg of TA powder with 1 mL of PEI aqueous solution (*M*_w_ = 1800, 10 000, or 70 000, 10 wt%), TA can connect to PEI chain through physical interactions such as electrostatic interaction and hydrogen bonding (blue circle in Figure 1a). Meanwhile, the hydrophobic 1,2‐dithiolane and alkyl chain of TA molecules can aggregate to form a hydrophobic domain (yellow circle in Figure 1a), resulting in the formation of PEI/TA complex microdroplets or macrofragments (red arrow) suspended in water (Figure 1b1; Figure S1, Supporting information). By increasing the *M*_w_ of PEI from 1800 to 70 000, the PEI/TA complex changed from microdroplets to macrofragments because the PEI with larger *M*_w_ can potentially form larger entangled domains (Figure 1b1; Figure S1, Supporting information). Second, the PEI/TA complex fragments aggregated to induce macroscopic fluid–fluid phase separation (dilute and dense phase) after gentle centrifugation or standing still over time. The dense phase, thereafter referred to as the PEI/TA coacervate, was a continuous phase (green fluorescence) containing vacuoles (yellow arrow in Figure 1b2). Finally, the polymer condensate further stabilized, possibly by continued aggregation of hydrophobic 1,2‐dithiolanes in TA molecules, leading to the transition from the PEI/TA coacervate to the PEI/TA hydrogel. Meanwhile, water exuded out from the coacervate due to the further aggregation of PEI/TA complex, resulting in more and larger vacuoles (yellow arrow) in the coacervate‐derived hydrogel (Figure 1b3).

With increasing *M*_w_ of PEI, the gelation time of PEI/TA coacervate decreased, while the storage modulus (*G′*) of the obtained PEI/TA hydrogel increased (Figure 1c,d). Moreover, the gelation time of PEI/TA coacervate significantly decreased with increasing centrifugation time (Figure S2, Supporting information). To balance the gelation time of PEI/TA coacervate and mechanical properties of PEI/TA coacervate‐derived hydrogel, the PEI with *M*_w_ of 10 000 and centrifugation time of 2 min (gelation time: 51 ± 12 s) were used in subsequent experiments. Because the gelation time of this coacervate is very short, we have developed a simple method to store this coacervate by mixing aqueous solution of PEI and TA powder and centrifugating the obtained PEI/TA suspension, which was then freeze‐dried immediately. Addition of water to the stored freeze‐dried PEI/TA coacervate powder resulted in the formation of coacervate upon hydration, and this coacervate can solidify into a hydrogel within minutes (Figure S3, Supporting information). Therefore, this coacervate could be stored by freeze‐drying and used after simple rehydration.

We next studied the cross‐linking mechanisms of PEI/TA coacervate and coacervate‐derived hydrogel and coacervate‐to‐hydrogel transition. As shown by the Fourier transform infrared (FTIR) spectra of PEI, TA, PEI/TA coacervate, and PEI/TA coacervate‐derived hydrogel (Figure 1e), the amine (—NH_2_ or —NH) peaks in PEI at 1583 cm^−1^ and the carboxylic acid (—COOH) peaks at 1690 cm^−1^ in TA shifted to 1560 and 1648 cm^−1^ in the PEI/TA coacervate, and then shifted to 1546 and 1628 cm^−1^ in the PEI/TA hydrogel, respectively. The shifting of the characteristic amine and carboxylic acid peaks in the PEI/TA hydrogel without the appearance of new characteristic peaks indicates that PEI and TA are cross‐linked through physical interactions such as hydrogen bonding and electrostatic interaction instead of covalent bonds. Moreover, the shifting of the characteristic amine and carboxylic acid peaks from coacervate to hydrogel also reflects the strong electrostatic interaction between PEI and TA upon the gelation. Besides, as shown by UV–vis spectra, the TA monomer, PEI/TA coacervate, and PEI/TA coacervate‐derived hydrogel showed obvious characteristic peaks at 325, 329, and 334 nm, which underwent an obvious redshift upon gelation. This redshift from 329 to 334 nm can be attributed to the aggregation of hydrophobic 1,2‐dithiolanes groups in TA molecules (Figure 1f).^[^ ^14^ ^]^ In addition, the obtained PEI/TA hydrogel can be completely dissolved in NaOH aqueous solution (1 mol L^−1^), due to the disruption of physical bonds between PEI and TA (Figure S4, Supporting information). These results indicate that the transition from coacervate to hydrogel is driven by the spontaneous hydrophobic aggregation of TA molecules and strong electrostatic interaction between PEI and TA, and the PEI/TA hydrogel is solely cross‐linked by physical interactions.

The PEI/TA coacervate‐derived hydrogel can be fabricated in custom free standing shapes and exhibited excellent self‐healing ability. We injected PEI/TA coacervate into a dumbbell shaped mold to form the corresponding hydrogel, demonstrating the potential of PEI/TA coacervate‐derived hydrogel to fit irregular‐shaped target wounds (Figure 1f; Movie S1, Supporting information). Moreover, upon surface contact, four individual PEI/TA hydrogels rapidly integrated to form a whole hydrogel, which was able to resist large stretching deformation (Figure 1g). The self‐healing capability can help maintain the integrity of the PEI/TA hydrogel‐based wound dressing under mechanical challenges.^[^ ^15^ ^]^ Besides, the solid content of PEI/TA coacervate‐derived hydrogel gradually decreased in simulated physiological fluid, indicating degradation of hydrogel in physiological environment (Figure S5, Supporting information). The degradation of PEI/TA coacervate‐derived hydrogel is important for its function as the wound dressing to facilitate wound healing.^[^ ^16^ ^]^ In addition, this PEI/TA coacervate‐derived hydrogel was stable against temperature rise up to 70 °C, which is the melting temperature of TA (Figure S6, Supporting information).^[^ ^12^ ^]^

Finally, we coincubated 3T3 cells with either PEI/TA coacervate‐derived hydrogel or positive control medium. After 24 h, over 95% of the cells remained viable with a spindle like morphology with no statistically significant difference between the two groups (Figure 1h). Free PEI is known to be toxic due to its strong positive charge.^[^ ^17^ ^]^ In this work, at the selected weight ratio of PEI and TA (1:2.2), the positive charge of PEI could be balanced by TA, resulting in a cytocompatible Zeta potential (0.2 ± 0.4 mV) and pH (7.0 ± 0.04) of the mixture (Figure S7, Supporting information). The charge balance between the positive charge of PEI and the negative charge of TA may have contributed to the observed low cytotoxicity of PEI/TA hydrogels.^[^ ^3^, ^18^ ^]^

### 2.2 Underwater Adhesiveness of PEI/TA Coacervate‐Derived Hydrogel

**Figure 2:**
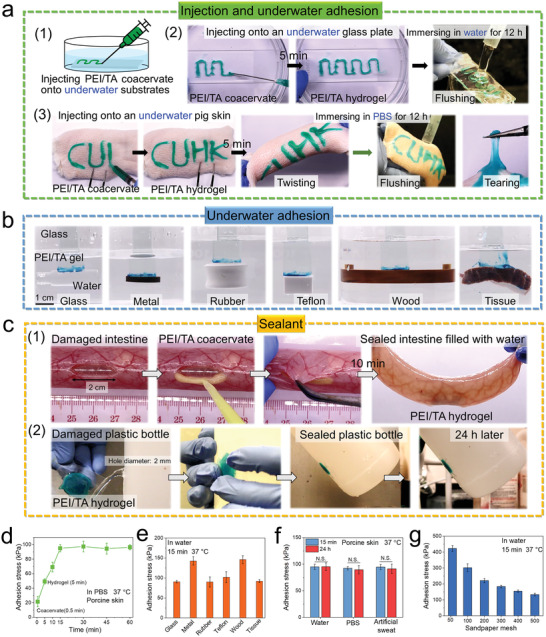
Robust adhesion of PEI/TA coacervate‐derived hydrogel on various underwater substrates. a) Schematic illustration of injecting PEI/TA coacervate onto an underwater glass plate (1), and strong adhesion of PEI/TA coacervate‐derived hydrogel in situ formed on the underwater glass plate (2) and porcine skin (3). b) Strong underwater adhesion of PEI/TA coacervate‐derived hydrogel on various substrates, such as glass, metal, rubber, Teflon, wood, and tissue. c) The PEI/TA coacervate‐derived hydrogel can effectively seal a damaged porcine intestine in vitro (1) and a leaky plastic bottle filled with water (2). d) Adhesion stress of PEI/TA coacervate‐derived hydrogel on underwater porcine skins at 37 °C after being immersed in PBS (*n* = 3). e) Adhesion stress of the PEI/TA hydrogel on various underwater substrates at 37 °C (*n* = 3). f) Adhesion stress of the PEI/TA hydrogel on porcine skins before and after incubation in water, PBS and artificial sweat at 37 °C for 24 h (*n* = 3). g) Adhesion stress of the PEI/TA hydrogel on underwater sandpapers with varying mesh (*n* = 3). Statistical significance was calculated by Student's *t*‐test. ^N.S.^ *P* > 0.05. Data are shown as the mean ± SD.

**Figure 3:**
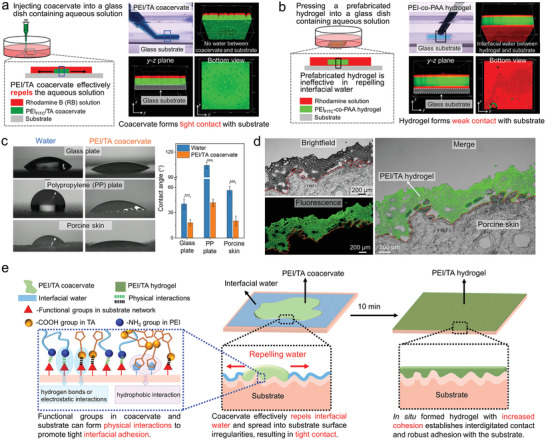
Underwater adhesion mechanism of PEI/TA coacervate‐derived hydrogel. a,b) Schematic illustration of injecting fluorescent PEI/TA coacervate (a) and pressing the pre‐fabricated PEI‐*co*‐PAA hydrogel (b) into the glass dish containing rhodamine aqueous solution (left), and the confocal microscopy images of the interface between coacervate/prefabricated hydrogel and substrate (right). c) Contact angles of water and PEI/TA coacervate on various substrates including hydrophilic glass plate, hydrophobic polypropylene, and porcine skin (*n* = 3). d) Microscopy images of cross‐section of in situ formed fluorescent PEI_FITC_/TA hydrogel adhered onto a piece of porcine skin. e) Schematic illustration of the putative underwater adhesion mechanism of PEI/TA coacervate‐derived hydrogel. Statistical significance was calculated by Student's *t*‐test. ***P < 0.001. Data are shown as the mean ± SD.

The PEI/TA coacervate‐derived hydrogel showed robust adhesion on various substrates underwater. We injected the PEI/TA coacervate onto an underwater glass plate and a piece of porcine skin, the PEI/TA coacervate transformed to the hydrogel in situ and tightly adhered onto the glass plate and porcine skin. After being immersed in aqueous solutions for 12 h, the in situ formed PEI/TA hydrogel still tightly adhered onto the glass plate and porcine skin without detachment despite water flushing or tearing (**Figure** **2**a; Movies S2 and S3, Supporting information). Moreover, various underwater substrates (such as glass, metal, rubber, Teflon, wood, and tissue) also can be glued to a glass block and lifted against their own weight by the PEI/TA coacervate‐derived hydrogels (Figure 2b; Movie S4, Supporting information). In addition, the PEI/TA coacervate‐derived hydrogel can be used as sealants. As shown in Figure 2c1, the PEI/TA coacervate‐derived hydrogel can seal a 2 cm‐long strip wound in a porcine intestine in vitro by depositing PEI/TA coacervate onto the margin of the wound followed by gelation. Ten minutes later, the intestine tissue can be tightly adhered together to hold water (Movie S5, Supporting information). We then adhered a polyvinyl chloride film with a layer of PEI/TA coacervate‐derived hydrogel onto a leaking plastic bottle (diameter of the hole was 7 mm) to successfully seal the leakage (Figure 3c2; Movie S6, Supporting information).

To study whether the PEI/TA coacervate‐derived hydrogel causes skin damage upon removal, we deposited PEI/TA coacervate onto the back of nude mice and removed the PEI/TA coacervate‐derived hydrogel by using a wet towel containing NaHCO_3_ aqueous solution. Hematoxylin and eosin (H&E) staining revealed no obvious difference in the skin structure between the experimental and control groups, indicating minimal skin damage due to the removal of hydrogel patch (Figure S8, Supporting information). The alkalescence of the NaHCO_3_ aqueous solution can break physical interaction between hydrogel and substrate as well as physical interactions in the hydrogel, resulting in the easy removal of PEI/TA coacervate‐derived hydrogel and minimal skin damage. Moreover, the PEI/TA coacervate‐derived hydrogel is suitable for one‐time use instead of repeatable or reusable adhesion. Because the PEI/TA coacervate can effectively repel interfacial aqueous solution and spread into surface irregularities of substrate to form tight contact with it, which plays a very important role in the adhesion of this hydrogel. However, after the transition from coacervate to hydrogel, the hydrogel is no longer capable of repelling interfacial aqueous solution and spreading into surface irregularities of substrate, resulting in nonadhesion of the prefabricated hydrogel to porcine skin (Figure S9, Supporting information).

Finally, we quantitatively evaluated the underwater adhesion stress of PEI/TA coacervate‐derived hydrogel on substrates. We first deposited the PEI/TA coacervate onto the underwater porcine skins and measured their adhesion stress at selected time points. From 30 s to 15 min, the adhesion stress of PEI/TA complex improved from 21.6 ± 7.0 kPa (coacervate) to 95.1 ± 4.9 kPa (hydrogel) due to the formation of more cross‐linking interactions and increasing cohesion of PEI/TA coacervate‐derived hydrogel (Figure S10, Supporting information). The adhesion stress of hydrogel subsequently remained stable from 15 to 60 min (Figure 2d). Moreover, the adhesion stresses of PEI/TA hydrogel on glass, metal, rubber, Teflon, wood, and tissue were 90.6 ± 2.8, 142.6 ± 10.3, 90.1 ± 12.6, 101.9 ± 13.7, 145.98 ± 9.8, and 92.2 ± 3.6 kPa, respectively, indicating their excellent adhesion on both hydrophilic and hydrophobic substrates (Figure 2e). Furthermore, after being immersed in water, PBS or artificial sweat for 24 h, the adhesion stress of PEI/TA hydrogel on porcine skins remained stable (Figure 2f). In addition, the underwater adhesion stress on sandpaper with varying grit has been compared. As shown in Figure 2g, adhesion stress increased from 133 to 422 kPa with the decreasing grit, indicating that the PEI/TA coacervate‐derived hydrogel can form stronger adhesion on rougher surfaces. For comparison, we listed the adhesion stresses of typical adhesives that can repel water to form adhesion on wet substrates in Table S1 of the Supporting information. This PEI/TA coacervate‐derived hydrogels showed a higher adhesion stress than most existing wet adhesives. Although hybrid protein‐like polyester coacervate‐derived adhesive shows a similar adhesion stress to PEI/TA coacervate‐derived hydrogel, it requires UV light irradiation to trigger cross‐linking of coacervate to achieve strong adhesion.

To study the underwater adhesion mechanism of PEI/TA coacervate‐derived hydrogel, we examined its ability to repel interfacial water by first injecting fluorescent PEI_FITC_/TA coacervate into a glass or polypropylene dish containing rhodamine aqueous solution (Movie S7, Supporting information) and then observing the interface between the coacervate and substrate with the confocal microscope. As shown in **Figure** **3**a and Figure S11 (Supporting information), there was no residual rhodamine aqueous solution between the coacervate and substrates, indicating that the coacervate can effectively repel water to form tight contact with the substrates. For comparison, a bulk polyethylenimine‐*co*‐poly(acrylic acid) (PEI‐*co*‐PAA) hydrogel that was prepared by redox‐initiated polymerization of acrylic acid (AA) in the presence of PEI and a chemical cross‐linker was pressed into the dishes, and a layer of red rhodamine solution was evidently observed between the hydrogel and substrates (Figure 3b; Figure S11, Supporting information). Then, we measured the contact angles of PEI/TA coacervate and water on hydrophilic glass plate, hydrophobic polypropylene plate, and porcine skin, respectively. The contact angles of the PEI/TA coacervate were all smaller than those of water on the selected substrates (Figure 3c), indicating that PEI/TA coacervate has a better wetting capability than water on these materials.^[^ ^2^, ^11^ ^]^ In addition, we deposited fluorescent PEI_FITC_/TA coacervate onto a piece of underwater porcine skin and kept them in PBS for 12 h. The sample was then sectioned into 20 µm slices across the hydrogel–tissue interface and observed with a confocal microscope. The in situ formed PEI/TA hydrogel completely filled up the porcine skin surface irregularities and formed congruent contact with the porcine skin (Figure 3d). Therefore, we believe that the putative underwater adhesion mechanism of PEI/TA coacervate‐derived hydrogel can be described as follows: upon contacting underwater or wet substrates, the PEI/TA coacervate can effectively repel interfacial aqueous solution and spread into surface irregularities of substrate to form tight contact with it; meanwhile, the amine groups in PEI, carboxyl acid groups, and 1,2‐dithiolanes and alkyl chain groups in TA can form physical interactions (such as hydrogen bonding, electrostatic interaction, and hydrophobic interaction) with functional groups on the substrate surface to induce tight interfacial adhesion on the substrates; the subsequent solidification of coacervate to hydrogel with increased cohesion establishes the interdigitated contact and robust adhesion with the substrate (Figure 3e).

### 2.3 Molecular Dynamics (MD) Simulations of the Formation and Substrate Adhesion of PEI/TA Coacervate‐Derived Hydrogel

**Figure 4:**
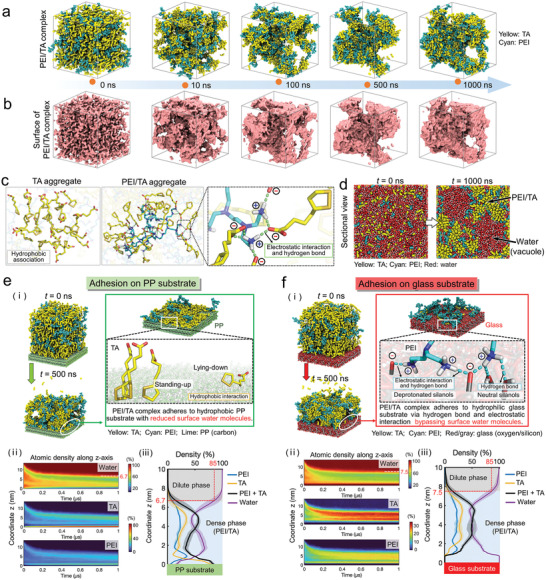
Simulations of the formation and substrate adhesion of PEI/TA coacervate‐derived hydrogel. a) The formation of PEI/TA hydrogel observed in microsecond MD simulations. b) Solvent‐accessible surfaces of the PEI/TA complex during MD simulations. c) Physical interactions within the PEI/TA complex (green dashed lines indicate electrostatic interaction and hydrogen bonds between PEI and TA). d) Sectional views of the PEI/TA self‐assembly system at *t* = 0 and *t* = 1000 ns. e,f) PEI/TA adhesion on PP and glass (PEI or TA within 0.3 nm are shown in the zoomed‐in panel. Dashed lines (cyan) represent hydrogen bonds formed between the substrate and PEI (cyan) or TA (yellow) (i), atomic density of PEI, TA and water along the *z*‐axis during the 1 µs simulations (ii), and the last 100 ns simulations (iii), respectively. Shaded areas show the standard deviation computed from the four replica simulations.

In order to uncover molecular details in the formation of PEI/TA coacervate and coacervate‐derived hydrogel, we next performed altogether 12 µs all‐atom MD simulations. First, four replicas of 1 µs self‐assembly simulations were carried out for a solvated system with 10.0 wt% PEI and TA in an initially random distribution. As shown in **Figure** **4**a, TA and PEI rapidly aggregated once the simulations began, leading to a suspension of PEI/TA clusters. This process appeared to involve two distinct types of aggregation, i.e., TA aggregation driven by hydrophobic association between their 1,2‐dithiolanes and alkyl chains, and PEI/TA aggregation with a core of a single PEI connected by hydrogen bonds to a shell consisting of multiple TAs driven by their electrostatic interaction (Figure 4c). Thereafter, the small PEI/TA clusters further aggregated to form a continuous coacervate phase in a liquid–liquid phase separation that began within 100 ns (Figure S12a, Supporting information). Throughout the rest of the simulations, the size of the coacervate phase remained largely unchanged, although internal polar interactions continued to increase gradually, reflecting subtle geometric adjustments within the coacervate phase (Figure S12b,c, Supporting information). Fitted with a biexponential equation, the number of polar cross‐linking between PEI and TA was found to converge to its final equilibrium value after ≈500 ns, ultimately leading to the formation of a continuous phase with vacuoles (Figure 1a,d; Figure S12b, Supporting information). Meanwhile, the solvent accessible surface area of the PEI/TA complex gradually decreased to ≈30.0% of the initial suspension, again reflecting the continuous structural rearrangement within the PEI/TA complex (Figure 4b; Figure S12d, Supporting information).

Next, we examined underwater adhesion of the PEI/TA complex on two substrates with different surface wettability, i.e., the hydrophobic polypropylene (PP) and the hydrophilic silicate glass substrates, through four replicate 1 µs MD simulations for each substrate. Notably, while PEI/TA adhesion was observed in all simulations, molecular details of such adhesion differ between these two substrates. For the hydrophobic PP surface, the interfacial hydrophobic association derived from the 1,2‐dithiolanes and alkyl chains of TA drove their assembly on top of PP and repelled interfacial water. A 1.0 nm TA layer covered 79.9% of the PP surface (Figure 4e(ii); Figure S13, Supporting information), where direct contacts were recorded between the hydrophobic moieties of TA and PP, with the former adopting either a “standing‐up” or “lying‐down” pose (Figure 4e(i)). Interestingly, PEI tended to aggregate on top of the TA layer, forming a PEI‐dominated composite layer of about 1.5 nm (Figure 4e(ii,iii)). The mobility of both PEI and TA, as quantified by their diffusion coefficients, was drastically reduced compared to that in the dilute phase (Figure S15, Supporting information). Collectively, the PEI/TA complex can form a hydrogel phase with stable adhesion on the PP substrate mainly through hydrophobic interaction (Figure 4e(iii)).

The glass substrate is hydrophilic in an aqueous environment due to the partial rehydration of surficial siloxanes into silanols, 5–20% of which are subsequently deprotonated under physiological conditions.^[^ ^19^ ^]^ MD simulations revealed that PEI made scattered direct contacts with the glass substrate bypassing the surficial water molecules (Figure 4f; Figure S14, Supporting information). Specifically, the PEI chains formed point contacts to the glass surface at a density of ≈0.1 nm^−2^ through the hydrogen bonds between their amines and both the neutral and deprotonated glass silanols (cyan dashed lines). The strong cooperative interaction of the hydrogen bonds with the electrostatic interactions between the protonated amines of PEI and the deprotonated silanols of the glass substrate further strengthened the anchorage of the former onto the latter (Figure 4f(i)). Indeed, once formed, these point contacts were remarkably stable, with the corresponding amines of PEI demonstrating only about a quarter of the mobility of the TA adhered onto the PP substrate (Figure S15, Supporting information). In an interesting contrast to the PP substrate, with PEI anchored on the glass surface, TA tended to aggregate on top of the PEI layer, forming a TA‐dominated composite layer of about 2.0 nm thickness (Figure 4f(ii,iii)). Collectively, the above physical interactions restrict the PEI/TA mobility, thereby stabilizing the adhesion of subsequently formed hydrogel on the glass substrate (Figure 4f(iii)).

### 2.4 Blood Repelling Capability and Protein Delivery of PEI/TA Coacervate‐Derived Hydrogel

**Figure 5:**
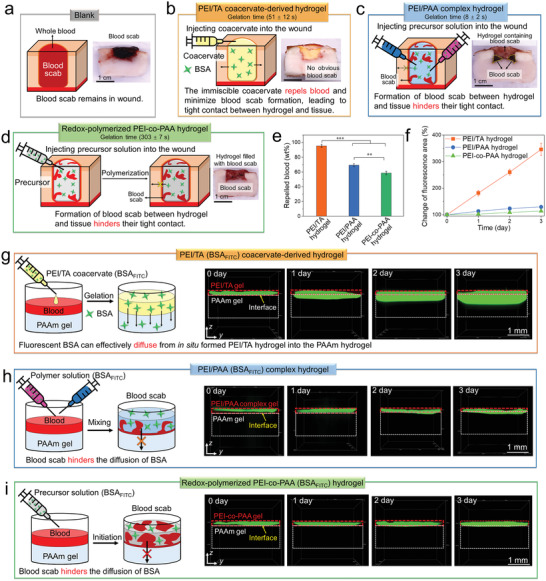
Effective blood repelling and delivery of cargo protein by the PEI/TA coacervate‐derived hydrogel. a–d) Schematic illustration and photos of porcine skin wounds containing blood with no treatment (a), and treated with the PEI/TA coacervate‐derived hydrogel (b), PEI/PAA complex hydrogel (c), or PEI‐*co*‐PAA hydrogel (d). The PEI/PAA complex hydrogel was prepared by mixing PEI and PAA solution, PEI‐*co*‐PAA hydrogel was prepared by redox‐initiated polymerization of acrylic acid (AA) in the presence of PEI and a chemical cross‐linker. The solid content of all hydrogels was kept at 56 wt%. e) Percentage of blood repelled by different hydrogels (*n* = 3). f) Change in the fluorescence area in the PAAm hydrogel substrate under different hydrogels (*n* = 3). g–i) Schematic illustration and confocal images of diffusion of fluorescent BSA from the PEI/TA coacervate‐derived hydrogel (g), PEI/PAA complex hydrogel (h), and PEI‐*co*‐PAA hydrogel (i) into the PAAm hydrogel substrate. Statistical significance was analyzed by one‐way ANOVA followed by a Tukey post hoc analysis between three groups, **P < 0.01 and ***P < 0.001. Data are shown as the mean ± SD.

To demonstrate the superior blood repelling capability of PEI/TA coacervate‐derived hydrogel (gelation time: 51 ± 12 s), two types of aqueous solution‐derived hydrogels including polyethylenimine/poly(acrylic acid) (PEI/PAA) complex hydrogel (gelation time: 8 ± 2 s) and redox‐polymerized PEI‐*co*‐PAA hydrogel (gelation time: 303 ± 7 s) were used for comparison (**Figure** **5**a–d). We created a wound (length = 10 mm, width = 10 mm, and depth = 5 mm) on a piece of porcine skin and injected 500 µL whole blood into the wound to simulate the skin wound in vitro. No treatment resulted in the wound filled with blood scab (Figure 5a). By contrast, injecting 500 µL PEI/TA coacervate showed minimal mixing with the blood and effectively repelled the blood, resulting in almost no blood scab formation between the in situ formed PEI/TA hydrogel and tissue (Figure 5b). For comparison, PEI/PAA complex hydrogel was formed in the wound by injecting and mixing PEI and PAA aqueous solutions, but the gelation was too rapid to repel blood, resulting in the blood scab formation between hydrogel and tissue (yellow dotted box) and inside the PEI/PAA complex hydrogel due to partial mixing of the precursor solution with blood (Figure 5c). Moreover, the PEI‐*co*‐PAA hydrogel was formed in the wound by injecting and redox‐polymerizing PEI and AA aqueous solution. Due to the slow gelation of this hydrogel, the precursor solution substantially mixed with the blood, resulting in the formation of blood scab inside the PEI‐*co*‐PAA hydrogel (Figure 5d). In addition, the PEI/TA coacervate‐derived hydrogel repelled almost all blood in the wound due to the immiscible nature of coacervate, whereas the PEI/PAA complex hydrogel and PEI‐*co*‐PAA hydrogel absorbed blood due to mixing of the precursor solutions with blood before gelation (Figure 5e). The absence of the blood scab enhances the seamless and interdigitated surface contact of the PEI/TA coacervate‐derived hydrogel with the wound site tissues.

We next studied the delivery of protein from different hydrogels to substrate in vitro. The bovine serum albumin labeled with FITC (BSA_FITC_) and polyacrylamide (PAAm) hydrogel were used as the model protein and substrate, respectively. We first prepared PAAm hydrogels in 24‐well plates and covered them with whole blood, and we then deposited PEI/TA coacervate, PEI and PAA aqueous solutions, and PEI and AA aqueous solutions loaded with BSA_FITC_ into the wells, respectively. After gelation, confocal microscopy images showed that in the PEI/TA coacervate‐derived hydrogel group, the fluorescent area in *y*–*z* plane increased significantly over time, indicating the effective delivery of BSA_FITC_ from the PEI/TA hydrogel into the PAAm hydrogel via diffusion (Figure 5g,f). By contrast, for the PEI/PAA complex hydrogel and PEI‐*co*‐PAA groups, the fluorescent area in *y*–*z* plane only increased slightly over time, indicating that the blood scab in the hydrogel and the hydrogel–substrate interface significantly hindered the diffusion and delivery of the BSA_FITC_ into the underlying substrate (Figure 5f,h,i). These findings together showed that the PEI/TA coacervate can effectively repel the blood and form tight contact with the substrate, thereby enhancing the delivery of carried proteinaceous cargoes to the substrate.

### 2.5 PEI/TA Coacervate‐Derived Hydrogel for Wound Healing

**Figure 6:**
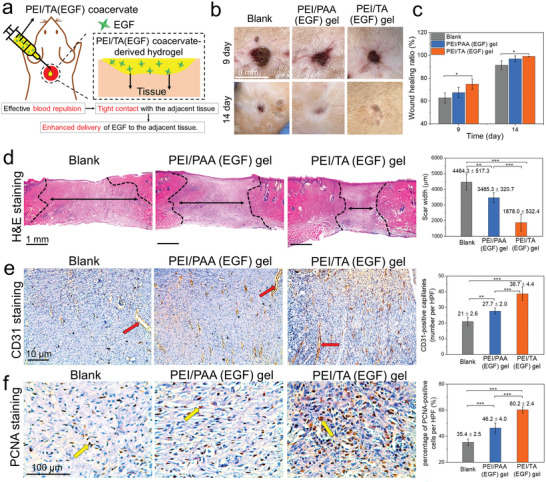
The PEI/TA coacervate‐derived hydrogel containing epidermal growth factor (EGF) enhances wound healing. a) Schematic illustration of treating full thickness skin wound with the PEI/TA (EGF) coacervate‐derived hydrogel. b,c) Photographs (b) and wound healing ratio (c) of skin wounds from different treatment groups on the day 9 and 14 after treatment. d) H&E staining images of wound site tissues (left) and scar width (black double‐headed arrows) of different groups (right). Black dashed lines show the outline of the neodermis regeneration and granulation tissue (original magnification × 20). e) Representative images of staining against the angiogenesis marker, CD31, at high power field (HPF, original magnification × 100, hematoxylin counterstain) (left) and CD31‐positive capillaries (red arrow) counts from images acquired at HPF (right). f) Representative images of staining against proliferating cell nuclear antigen (PCNA) at high power field (HPF, original magnification × 400, hematoxylin counterstain) (left) and the percentage of PCNA‐positive cells (yellow arrow) in images acquired at HPF (right). Rats, *n* = 6. Statistical significance was analyzed by one‐way ANOVA followed by a Tukey post hoc analysis between three groups, **P < 0.01 and ***P < 0.001. Data are shown as the mean ± SD.

The PEI/TA coacervate‐derived hydrogel was further used to repair full‐thickness skin wounds in vivo (**Figure** **6**a). Full thickness skin wounds (diameter: 8 mm) were created on the back of the rats and subsequently covered with the PEI/TA coacervate‐derived hydrogel containing EGF or PEI/PAA complex hydrogel containing EGF. The wound without any treatment was used as a control group. As shown in Figure 6b,c, the wound treated with the PEI/TA (EGF) hydrogel showed higher macroscopic healing ratio (74.7% ± 4.6%) compared to that of the wound treated with the PEI/PAA (EGF) hydrogel (67.4% ± 4.6%) and blank group (no treatment) (62.7% ± 4.4%) on day 9. On day 14, the wounds treated with the two hydrogels but not the blank group were nearly closed. We further used H&E staining to assess the healing of the wounds (Figure 6d). The wounds treated with the PEI/TA (EGF) hydrogel showed the smallest scar width (black double‐headed arrow) compared to the blank group (*P* < 0.001) and PEI/PAA (EGF) hydrogel group (*P* < 0.001).

We further evaluated the regenerated tissue of different groups through immunohistochemical staining against CD31 (a marker for endothelial cells and angiogenesis) and proliferating cell nuclear antigen (PCNA, a proliferation marker of G1/S phase). CD31 staining showed that the number of CD31‐positive capillaries (red arrow) in regenerated tissue of the PEI/TA (EGF) hydrogel group was significantly larger than that in the blank group (*P* < 0.001) and PEI/PAA (EGF) group (*P* < 0.001) (Figure 6e). The re‐epithelization in regenerated tissue showed that the percentage of PCNA‐positive cells (yellow arrow) in the PEI/TA (EGF) hydrogel group was significantly higher compared with that of blank group (*P* < 0.001) and PEI/PAA (EGF) group (*P* < 0.001) (Figure 6f). Taken together, the PEI/TA (EGF) coacervate can effectively repel blood and other body fluid at the wound sites to form tight contact with the adjacent tissue, leading to the enhanced delivery of EGF to the adjacent tissue and wound healing outcomes.

## 3 Conclusion

In this work, we demonstrated that based on the physical interactions between PEI and TA as well as the hydrophobic interactions between TA, the PEI/TA coacervate can be obtained by simply mixing PEI aqueous solution with TA powder. Upon being deposited onto underwater substrates of diverse properties, the PEI/TA coacervate can effectively repel interfacial water and completely spread into surface irregularities of substrates to form tight contact with them. Meanwhile, the functional groups between the coacervate and substrate can form physical interactions to further promote tight interfacial adhesion. The physical interactions between PEI and TA as well as hydrophobic aggregation of TA molecules enhance their cross‐linking and the cohesion of the coacervate, leading to the transition from coacervate to hydrogel. The in situ formed hydrogel can interlock itself with the substrates to establish robust wet adhesion despite substantial mechanical challenges. Owing to such excellent wet adhesive property, the PEI/TA coacervate‐derived hydrogel can effectively seal the damaged porcine intestine and leaky plastic bottle despite mechanical challenges and irregular surfaces. In addition, the PEI/TA coacervate‐derived hydrogel loaded with EGF can effectively repel wound site blood to form tight contact with surrounding tissues, thereby enhancing the delivery of EGF to the surrounding tissues. The excellent underwater adhesion, body fluid‐immiscibility, and easy preparation of PEI/TA coacervate‐derived hydrogel make it an ideal bioadhesive with promising potential in diverse biomedical applications such as wearable devices, sealants, and drug delivery.

## 4 Experimental Section

### Materials

PEI (*M*_w_ = 1800, 10 000, and 70 000) and TA were purchased from Aladdin (China). FITC was purchased from J&K Scientific (China). PEI_FITC_ was prepared by mixing 1 g PEI with 10 mg FITC in a mixed solution of DMSO and water at room temperature in the dark for 24 h. The mixture was purified in poor solvent acetone.

### PEI/TA Coacervate and Hydrogel Preparation

Adding TA powder (220 mg) into 1 mL 10 wt% PEI solution and mixing for 1–4 min, the PEI/TA suspension could be obtained. Then, the PEI/TA coacervate was obtained after being centrifuged at 10 000 r min^−1^ for 30–180 s. Next, the PEI/TA coacervate was directly injected into the mold to prepare correspondingly shaped hydrogel without any other operation. The PEI_FITC_ was used to prepare fluorescent PEI_FITC_/TA suspension, coacervate, and hydrogel, which were observed with a confocal microscope. The blue dye was occasionally added into PEI aqueous solution to prepare coacervate and hydrogel.

### Mechanical Tests

Dynamic viscoelasticity of coacervate and hydrogel was measured using a Kinexus rheometer with an 8 mm diameter flat plate. The oscillatory time sweep experiments were conducted under a fixed strain of 1.0% and a frequency of 0.5 Hz at 37 °C in PBS. Moreover, a lap shear test was performed using a Kinexus rheometer with custom clamps at a crosshead speed of 3 mm min^−1^. For the lap shear test, PEI/TA coacervate‐derived hydrogel was sandwiched between two substrates in water with an adhesion area of 2 cm × 1 cm. Then, the samples were incubated at 37 °C for different time with pressing (8 kPa pressure applied). The adhesion stress was calculated as follows: Adhesion stress = *F*_max_/(*wl*), where *F*_max_ was the maximum force, and *w* and *l* were the width and length of the adhesion area. Three specimens per condition were tested to ensure the reliability of the data.

### Contact Angles Test

PEI/TA coacervate and water were dropped onto glass plate, polypropylene plate, and porcine skin, respectively, which were recorded and measured by using a contact angle meter.

### Substrate Integration Capacity Test

Fluorescent PEI/TA coacervate or pressed bulk fluorescent PEI‐*co*‐PAA hydrogel was injected into the glass and polypropylene dishes containing rhodamine aqueous solution, and then observed them by using a confocal microscope. In addition, PEIFITC/TA was deposited onto a piece of underwater porcine skin, and kept in PBS for 12 h. Afterward, the samples were washed with PBS and frozen in OCT for 30 min. The frozen samples were sectioned into 20 µm slices along a cross‐section and then fixed onto glass slides for observation and imaging with a confocal microscope. In addition, PEI/TA coacervate, PEI/PAA complex hydrogel precursor solution, and PEI‐*co*‐PAA precursor solution were injected into the wounds containing blood in the porcine skins. After gelation, the samples were kept in −20 °C for 4 h, and then the cross‐sections of these samples were observed. PEI/PAA complex hydrogel was prepared by mixing PEI and PAA solution. PEI‐*co*‐PAA hydrogel was prepared by free redox polymerization of AA in the presence of PEI and a chemical cross‐linker. The solid content for all hydrogels were 56 wt%. Moreover, the gelation time of different hydrogels was tested with a Kinexus rheometer.

### MD Simulation Details

All MD simulations were carried out with GROMACS‐2020.6^[^ ^20^ ^]^ at all‐atom resolution for 1 µs in four replicas per system. The parameters of silicate glass were derived from INTERFACE force field (IFF),^[^ ^19^ ^]^ whereas all the other parameters were derived from CHARMM36^[^ ^21^ ^]^ and CHARMM General force field (CGenFF).^[^ ^22^ ^]^ Branched PEI chain was modeled within Avogadro^[^ ^23^ ^]^ based on the chemical structure bearing a molecular weight of 1800 and a net charge of 20, whereas TA was retrieved from the PubChem database^[^ ^24^ ^]^ with its carboxyl group deprotonated. PP substrate was constructed by compact stacking of isotactic PP chains, in which periodic molecule conditions were applied to prevent dangling ends. These structures were initially parameterized using the CGenFF server^[^ ^25^ ^]^ and then refined with Gaussian^[^ ^26^ ^]^ and the force field toolkit plugin^[^ ^27^ ^]^ of VMD.^[^ ^28^ ^]^ Silicate glass substrate with 9% of the surficial silanols deprotonated was modeled and parameterized based on the Q3 amorphous silica model under physiological conditions from the IFF database.^[^ ^19^ ^]^ All modeled structures were relaxed in water using NPT equilibrations beforehand to obtain reasonable geometries. Further details of the simulations are provided in the Supporting Information.

### In Vitro Blood Repulsion and Diffusion of BSA

A wound (length = 10 mm, width = 10 mm, and depth = 5 mm) was created on a piece of porcine skin, and 500 µL blood was added into the wound. Different materials (500 µL) were added into the wound containing blood, and the repelled blood was absorbed by using filter paper, which were used to record the weight of the repelled blood. The porcine skins samples were stored at −20 °C for 4 h, then the cross‐section of the samples was observed.

To observe the diffusion of the fluorescent BSA, the transparent PAAm hydrogels prepared in 24‐well plate were used as substrate. Different hydrogels containing fluorescent BSA were deposited into the well containing PAAm hydrogel that were covered with blood (200 µL). At preset time, the samples were observed by using confocal microscope.

### In Vivo Wound Healing Evaluation

Female rats with an average weight of 180–200 g were used in this study. Full skin wounds were created with 8 mm diameter on back of rats, and then covered these wounds with PEI/TA coacervate‐derived hydrogel containing EGF and PEI/PAA (EGF) hydrogel, respectively. The wounds without treatment were used as control group. Six rats for each group. To record the wound healing process, the wounds were recorded by taking photos on day 0, 9, and 14 after treatment. And the wound healing ratio was calculated as follows: Wound healing ratio = (*A*_0_ – *A*)/*A* × 100%, where *A*_0_ and *A* are the area of initial wound and current wound, respectively. Rats were euthanized 14 days after treatment, and the tissues were fixed in phosphate‐buffered formalin for 24 h, dehydrated, and embedded in paraffin. Paraffin‐embedded samples were cut into 7 µm sections, which were deparaffinized by using xylene and dehydrated using gradient alcohol. Then, the sections were stained with H&E. Moreover, immunohistochemistry was performed to detect PCNA (a cell proliferation marker) and assess regenerated blood vessels (CD31) in the wound according to the Supporting Information. Quantification of CD31 and PCNA expression was conducted in a blinded manner. Animal experiments were conducted according to the guidelines of the Animal Ordinance (Chapter 340), Department of Health, Hong Kong. The study was approved by the Animal Experimentation Ethics Committee of the Chinese University of Hong Kong (17‐145‐ITF).

### Statistical Analysis

Unless otherwise specified, all data were shown as means ± SD via at least triplicate samples. Independent Student's *t*‐test and one‐way ANOVA followed by a Tukey post hoc analysis were used to determine statistical significance between two or multiple groups, respectively. Statistical analyses were performed using SPSS (Statistical Package for the Social Sciences) 25.0, and a two‐sided *P* < 0.05 was considered statistically significant.

## Conflict of Interest

The authors declare no conflict of interest.

## Supporting information

Supporting Information

Supplemental Movie 1

Supplemental Movie 2

Supplemental Movie 3

Supplemental Movie 4

Supplemental Movie 5

Supplemental Movie 6

Supplemental Movie 7

## Data Availability

The data that support the findings of this study are available from the corresponding author upon reasonable request.
