# Supplementary material for: Coacervate‐Derived Hydrogel with Effective Water Repulsion and Robust Underwater Bioadhesion Promotes Wound Healing
Source: Adv Sci (Weinh). 2022 Sep 15;9(31):2203890. doi: 10.1002/advs.202203890 (PMC9631067; doi:10.1002/advs.202203890)
Supplement: Supplementary file 1 — Supporting Information [file ADVS-9-2203890-s003.pdf]

ADVANCED SCIENCE
Open Access

Supporting Information

for *Adv. Sci.*, DOI 10.1002/advs.202203890

Coacervate-Derived Hydrogel with Effective Water Repulsion and Robust Underwater Bioadhesion Promotes Wound Healing

Xin Peng, Yuan Li, Tianjie Li, Yucong Li, Yingrui Deng, Xian Xie, Yi Wang, Gang Li* and Liming Bian**

Supporting Information

Coacervate-Derived Hydrogel with Effective Water Repulsion and Robust Underwater Bioadhesion Promotes Wound Healing

Xin Peng, Yuan Li, Tianjie Li, Yucong Li, Yingrui Deng, Xian Xie, Yi Wang, Gang Li*, Liming Bian**

Experiment sections

Immunohistochemistry: Samples were firstly dewaxed using xylene and dehydrated by using gradient alcohol. After antigen retrieval with citrate buffer (10 mmol/L, pH 6.0) and inhibition of endogenous peroxidase activity with 3.0% hydrogen peroxide, non-specific binding was blocked by incubation in 1% bovine serum albumin for 1 h at room temperature. Then, the samples were incubated with the following primary antibodies at 4 °C overnight: anti-PCNA mouse polyclonal antibody, anti-CD31 mouse polyclonal antibody. After incubating with goat anti-mouse IgG, the samples were incubated with peroxidase-labeled streptavidin for 1 h and they were stained using diaminobenzidine (DAB) solution under microscopic control. Finally, the samples were stained with hematoxylin.

Molecular dynamics simulations details: For self-assembly simulations, 64 PEI (10.0 wt%) and 1,280 TA were randomly placed in a cubic box with a volume of 13.4 × 13.4 × 13.4 nm^3, and subsequently solvated with TIP3P water. The self-assembly system was subjected sequentially to energy minimization,1-ns NVT equilibration and 1-ns NPT equilibration. Thereafter, four copies of 1-μs production simulations were carried out in the NPT ensemble. For adhesion simulations, 64 PEI (10.0 wt%) and 1,280 TA were randomly placed over the substrate in a rectangular box with a lateral xy dimension of 12.9 × 13.8 nm^2 for PP and 13.9

× 13.5 nm^2 for glass and solvated with TIP3P water. A 20.0-nm vacuum slab and a flat-bottom potential restraint was established on top of the solvated system to avoid artificial interactions across the periodic boundary along the z axis. Specifically, an extended flat-bottom potential restraint was applied to the solvents to retain a 1.6-nm water-capped buffer at the top of the systems, preventing direct contacts of the solvated PEI and TA with the vacuum slab. The systems were first subjected to energy minimization and 1-ns NVT equilibration. Thereafter, four copies of 1-μs production simulations were carried out for each system in the NVT ensemble.

In all simulations, van der Waals forces were smoothly switched off from 0.8 nm to 0.9 nm. Electrostatics were calculated using the particle mesh Ewald (PME) method[1] with a cutoff of 0.9 nm. The systems were coupled at a temperature of 300 K with a velocity-rescaling thermostat[1], and a pressure of 1 bar with a Berendsen barostat[2]. All bonds containing hydrogen atoms were constrained using the LINCS algorithm[3]. All visualizations were conducted and rendered by VMD and PyMOL[4]. VMD was also used to calculate the atomic density and occupancy, as well as the intermolecular hydrogen bonds between PEI and TA within the PEI/TA phase, and between PEI/TA complex and substrate. The displacement of particles was measured with a 0.1-ns time lag. The diffusion coefficient was derived via the Einstein relation from the mean squared displacement (MSD), which was calculated with the center of mass of each moiety. Additional 100 1-ns replica simulations of each adhesion system initiated from their structures at t = 0 ns or t = 1000 ns were further conducted to obtain enough sampling for the calculation of diffusion coefficients. GROMACS was used to measure the size and solvent accessible surface area of the PEI/TA phase. The contact areas of PEI, TA and water on the substrate surface were obtained by extracting the corresponding portions from the solvent accessible surface areas.

Supporting Figures

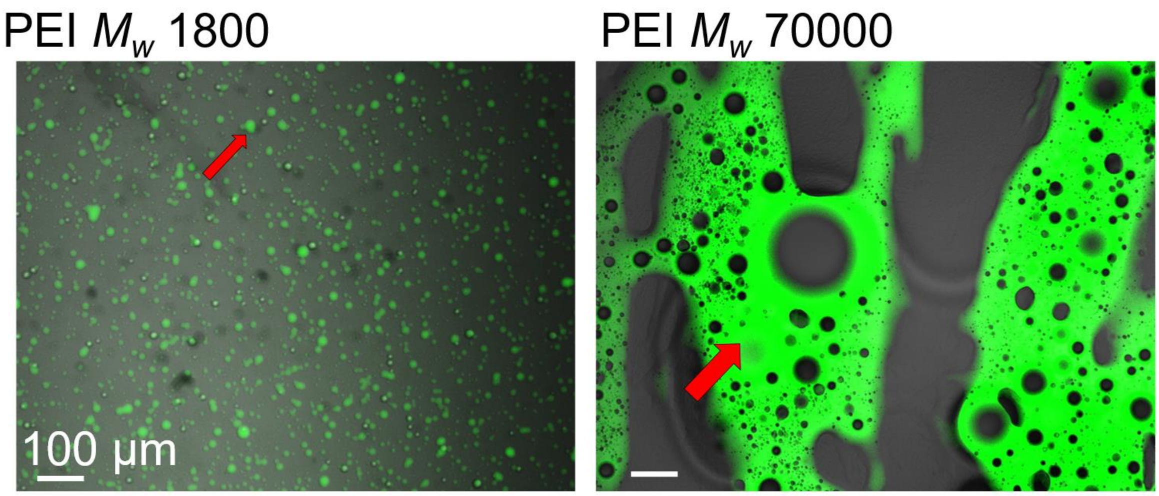

Figure S1. Macrograph of PEI/TA complex micro-droplets (left, M_w of PEI is 1800) and macro-fragments (right, M_w of PEI is 70000). Red arrow: PEI/TA complex. Macrograph are the merge of bright and fluorescent images.

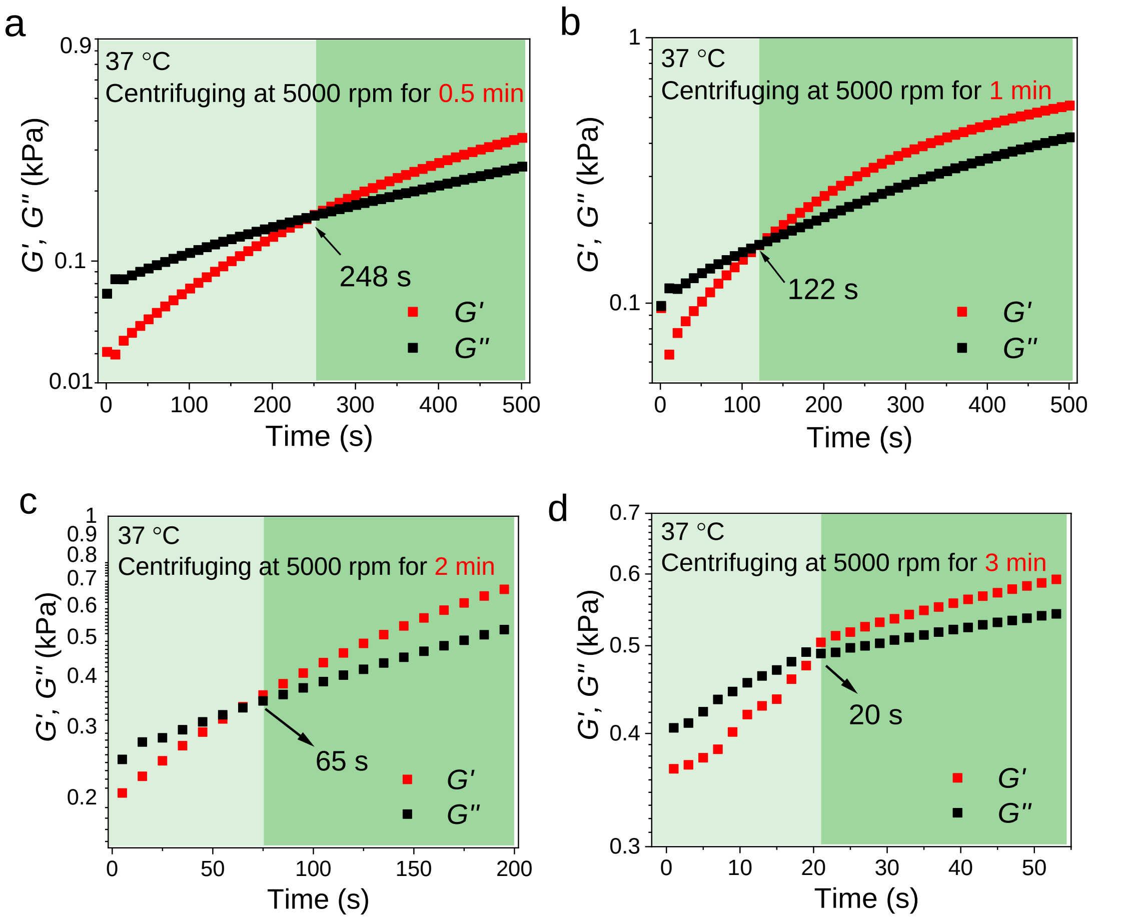

Figure S2. Gelation time of PEI/TA (M_w of PEI is 10000) coacervate prepared by centrifuging PEI/TA suspension at 10000 r/min for different time. Test Conditions: 37 °C in PBS.

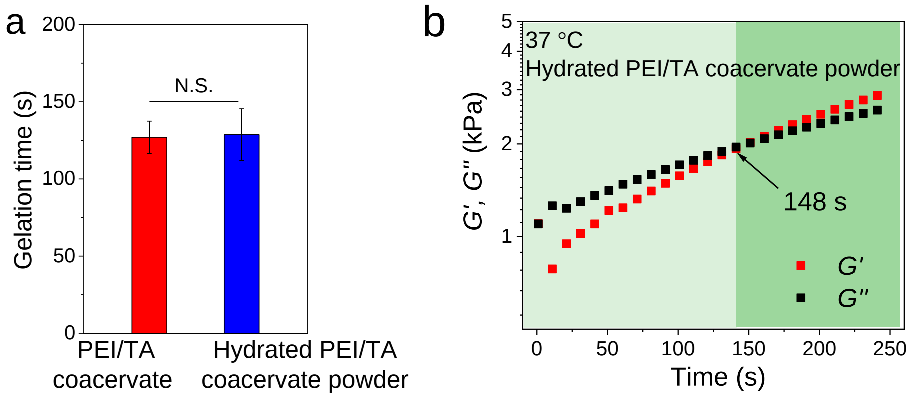

Figure S3. (a) Gelation time of original coacervate and hydrated PEI/TA coacervate powder. (b) Rheological test of hydrated PEI/TA coacervate powder. Test Conditions: 37 °C in PBS (n = 3). PEI/TA coacervate was prepared by centrifuging PEI/TA (M_w of PEI is 10000) suspension at 10000 r/min for 1 minute. Data are shown as the mean ± SD. Statistical significance was calculated by Student's t test. $^{N.S.}P > 0.05$.

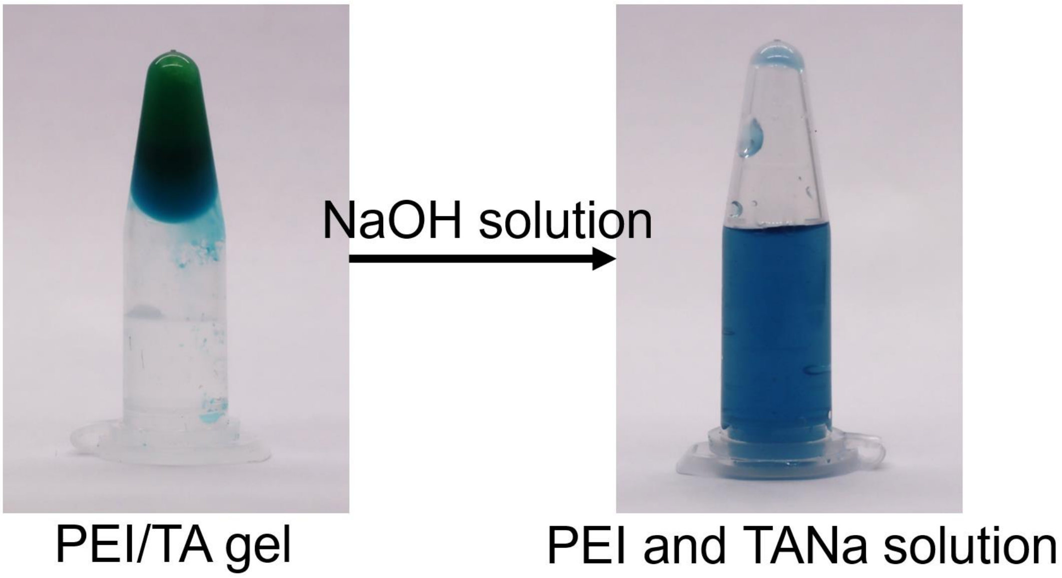

Figure S4. Dissolution of PEI/TA hydrogel in NaOH solution (1 mol L^{-1}).

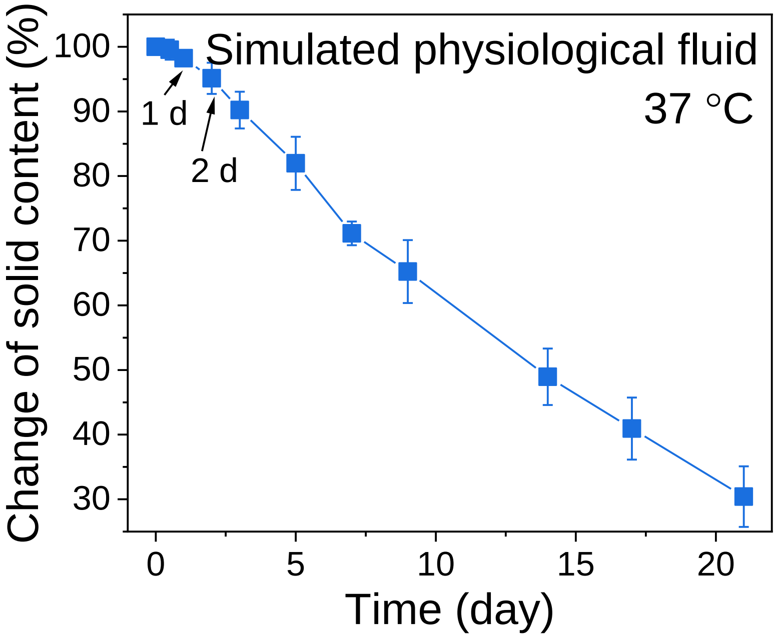

Figure S5. Change of the solid content of PEI/TA coacervate-derived hydrogel in simulated physiological fluid (SPF) at 37 °C (n = 3). Data are shown as the mean ± SD.

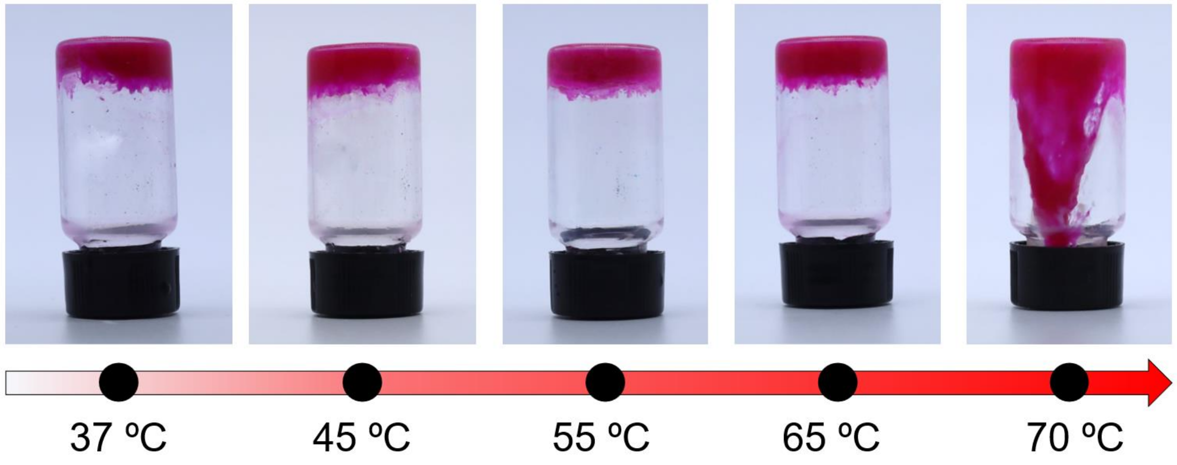

Figure S6. Changes of stability of the PEI/TA coacervate-derived hydrogel against temperature rise.

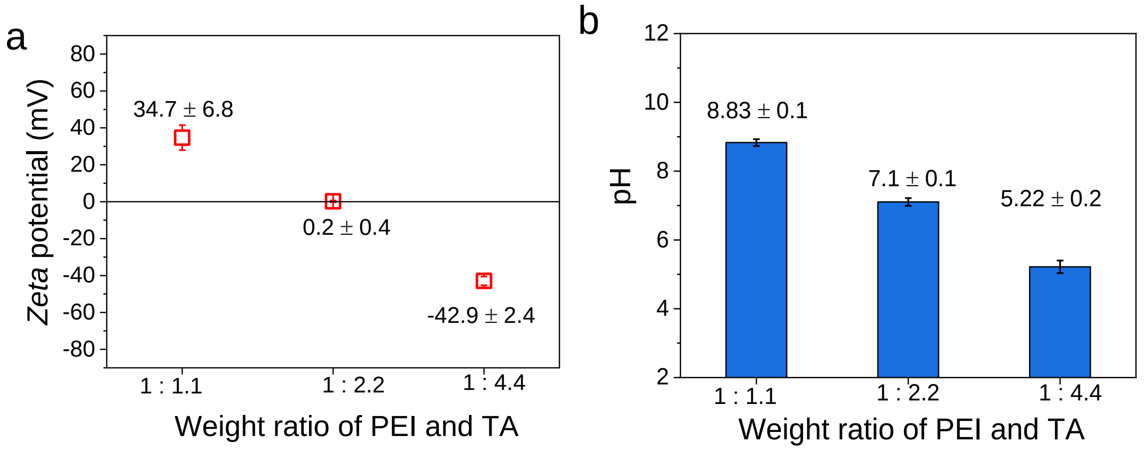

Figure S7. *Zeta* potential (a) and pH (b) of the PEI/TA hydrogels (50 mg) prepared with varying weight ratios of PEI and TA in deionized water (5 mL) (n = 3). Data are shown as the mean ± SD.

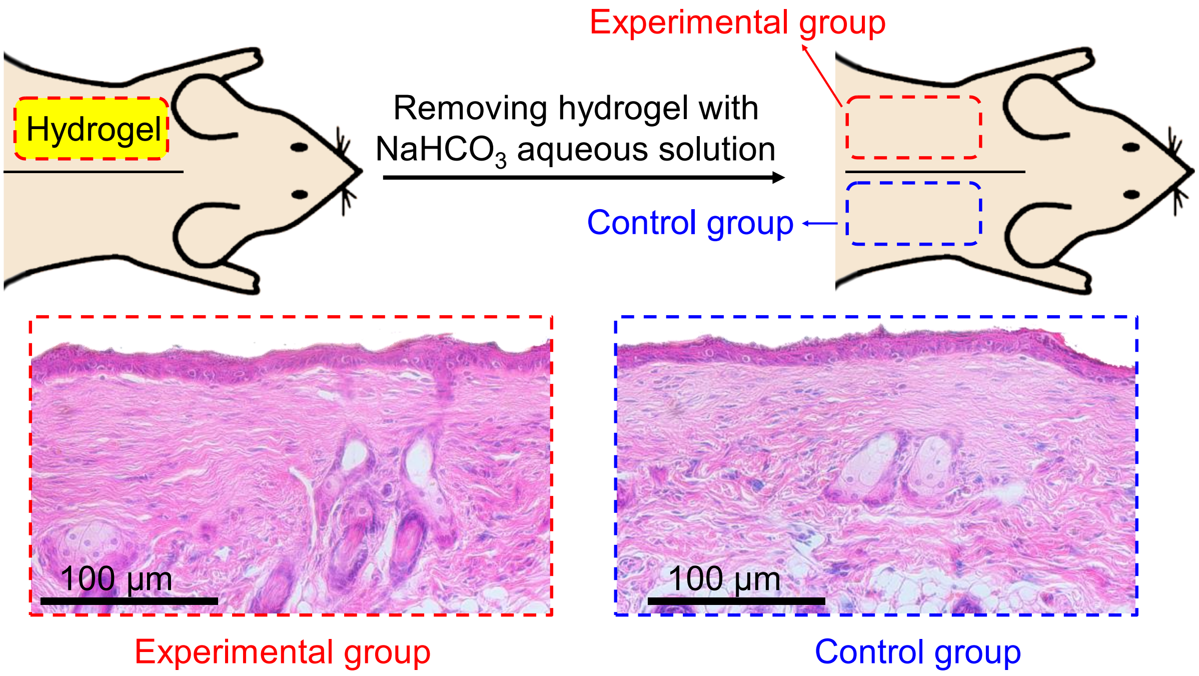

Figure S8. H&E staining of the skin after removing a PEI/TA coacervate-derived hydrogel with a wet towel containing $NaHCO_3$ aqueous solution (left) and the normal skin (right).

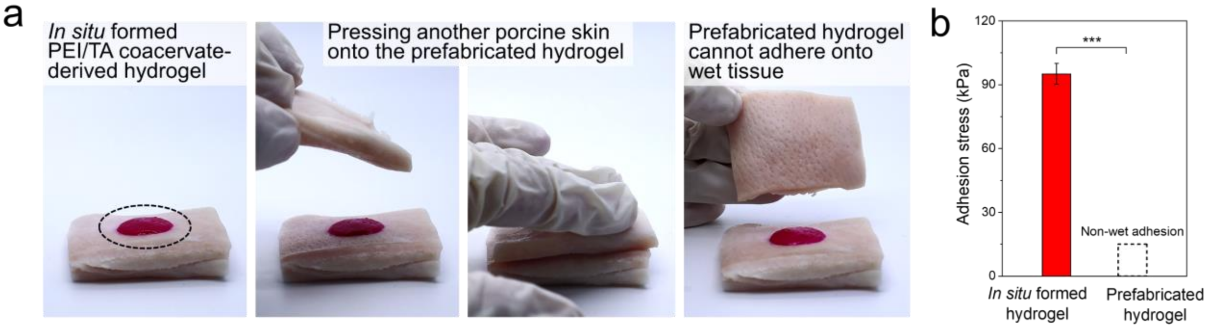

Figure S9. (a) Non-adhesion of prefabricated PEI/TA coacervate-derived hydrogel to wet porcine skin. (b) Adhesion stress of *in situ* formed PEI/TA coacervated-derived hydrogel and prefabricated hydrogel (n =3). Data are shown as the mean ± SD. Statistical significance was calculated by Student's t test. ***$P < 0.001$.

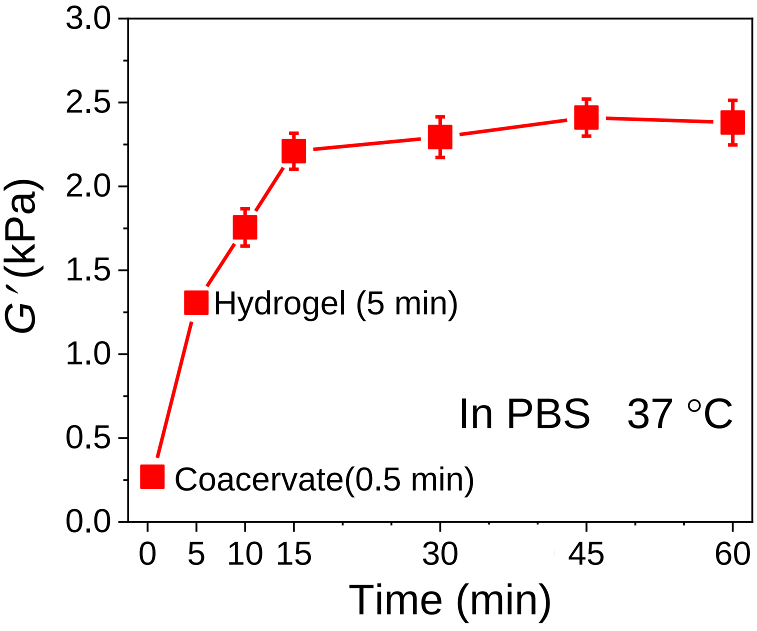

Figure S10. Change of Elastic modulus (G') of the PEI/TA complex over time. Data are shown as the mean ± SD.

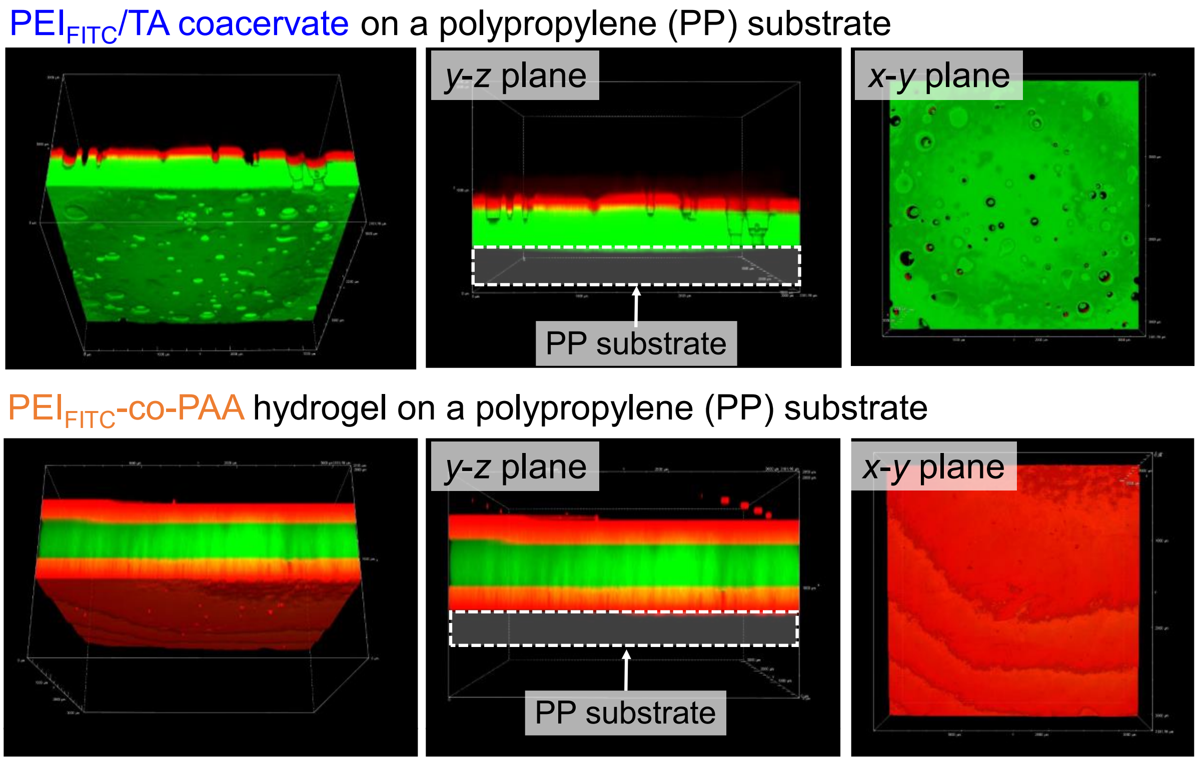

Figure S11. Confocal microscopy images of the interface between coacervate (above) / hydrogel (bottom) and polypropylene substrate.

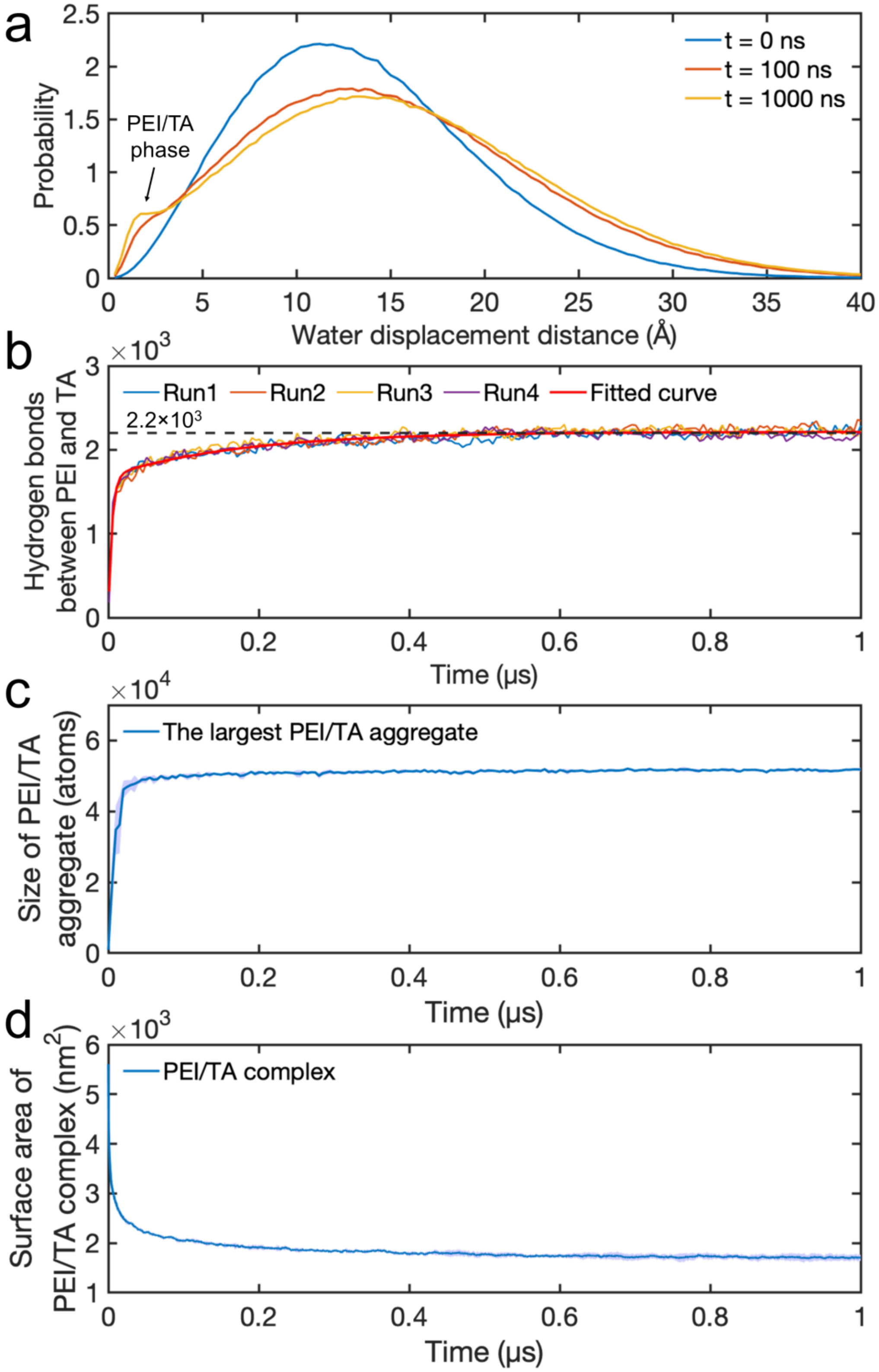

Figure S12. PEI/TA self-assembly simulations. (a) Probability of water displacement with a lag time of 0.1 ns showing the separation of a dense PEI/TA phase with slow water diffusion from the dilute solution phase at 100 ns and 1000 ns. (b) Number of hydrogen bonds between PEI and TA. The curve was fitted with a biexponential function (R^2 = 0.982). (c) The size of the largest PEI/TA aggregate. (d) The surface area of the PEI/TA complex. The shaded area shows the standard deviation computed from the four replica simulations.

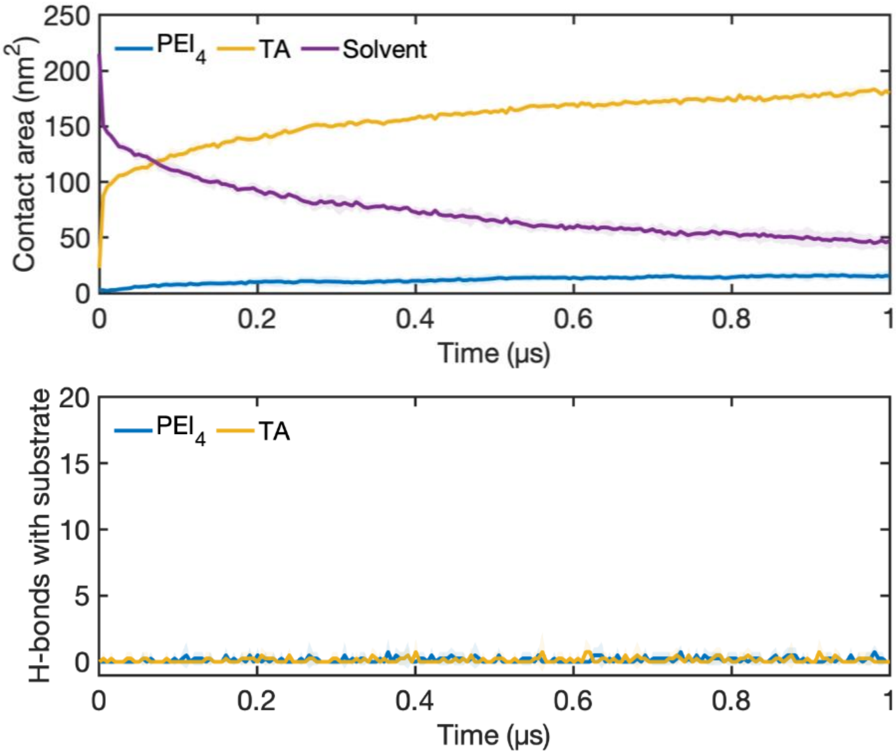

Figure S13. Contact areas and the number of hydrogen bonds (H-bonds) at the top interface of the substrate during MD simulations of PEI/TA adhesion on the hydrophobic PP surface. The shaded area shows the standard deviation computed from the four replica simulations.

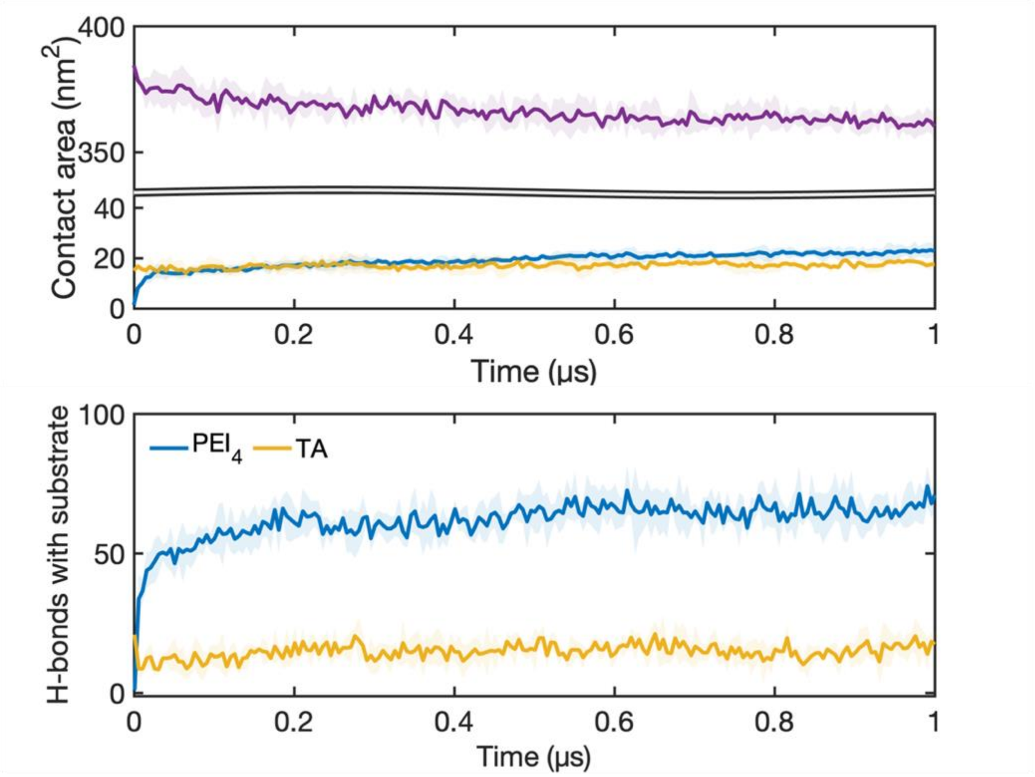

Figure S14. Contact areas and the number of hydrogen bonds (H-bonds) at the top interface of the substrate during MD simulations of PEI/TA adhesion on the hydrophilic glass surface. The shaded area shows the standard deviation computed from the four replica simulations.

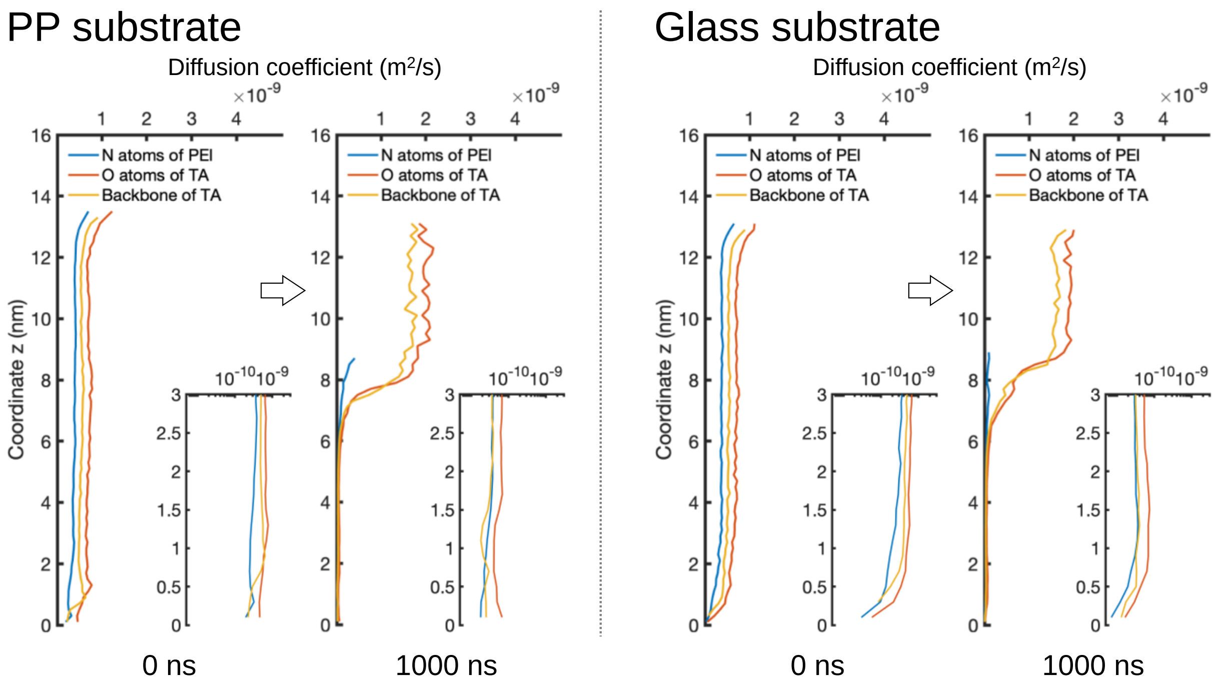

Figure S15. Diffusion coefficients of typical moieties of PEI and TA on PP and glass substrates at t = 0 and t = 1000 ns of MD simulations, with the zoomed-in panels showing the portion nearby the substrate in logical axes. The diffusion coefficients are derived via the *Einstein* relation from the mean squared displacement of typical moieties, which is proportional to their mobility.

Table S1. The adhesion stresses of the typical proposed adhesives that can repel water to form adhesion on wet substrates.

Adhesives		Substrate	Adhesion strength	Reference
**Coacervate-derived adhesives**	PEI/TA coacervate-derived hydrogel	Wet tissue	92 kPa	This work
	Hybrid protein-like polyester coacervate-derived adhesive	Wet glass	96 kPa	*ACS Nano*, **2020**, 14, 8359-8367.
**Coacervate**	Small molecule assembled nonionic coacervate	Wet tissue	3 kPa	*Adv. Funct. Mater.*, **2022**, 32, 2110320.
	Polyelectrolyte coacervate	Wet tissue	42 kPa	*Adv. Funct. Mater.*, **2022**, 32, 2105464.
	Nanoparticle-assembled bioadhesive coacervate	Wet tissue	41 kPa	*Nat. Commun.*, **2021**, 12, 7162.
	Hyperbranched polymer coacervate	Wet glass	69 kPa	*Adv. Mater.*, **2019**, 31, 1905761.
**Hydrogel**	Self-hydrophobic hydrogel	Wet glass	36 kPa	*Adv. Funct. Mater.*, **2020**, 30, 1907064.
**Hydrophobic fluid**	Multilayer adhesive patch containing hydrophobic fluid	Wet tissue	56 kPa	*Adv. Mater.*, **2021**, 33, 2007667.
	Adhesive containing hydrophobic oil matrix and bioadhesive microparticles	Wet tissue	70 kPa	*Nat. Biomed. Eng.*, **2021**, 5, 1131-1142.

Reference

1. Darden T., York D., Pedersen L. Particle mesh Ewald: An *N*·Log(*N*) method for Ewald sums in large systems. *J. Chem. Phys.* 98, 10089-10092 (1993).
2. Bussi G., Donadio D., Parrinello M. Canonical sampling through velocity rescaling. *J. Chem. Phys.* 126, 014101 (2007).
3. Berendsen H. J. C., Postma J. P. M., Gunsteren W. F. v., DiNola A., Haak J. R. Molecular dynamics with coupling to an external bath. *J. Chem. Phys.* 81, 3684-3690 (1984).

4. Hess B., Bekker H., Berendsen H. J. C., Fraaije J. G. E. M. LINCS: A linear constraint solver for molecular simulations. *J. Comput. Chem.* 18, 1463-1472 (1997).
5. Schrodinger, LLC. The PyMOL Molecular Graphics System, Version 2.0. (2015).
